# Catatonia in an Adolescent: Efficacy of ECT Following Partial Response to Benzodiazepines

**DOI:** 10.1192/bjo.2026.11794

**Published:** 2026-06-30

**Authors:** Mohammed Mehdi Hassan Meah, Chris Popoola, Barbara Geller, Praveen Kumar

**Affiliations:** 1 NHS Tayside, Dundee, United Kingdom; 2 University of Dundee, Dundee, United Kingdom; 3 Imperial College London, London, United Kingdom; 4 NHS Grampian, Aberdeen, United Kingdom

## Abstract

**Aims:**

Catatonia is a syndrome characterised by decreased, increased, or abnormal psychomotor activity and is seen in conditions such as schizophrenia, and depression. Global meta-analysis carried out in 2017 found mean catatonia prevalence to be 9.0%. This case involves an adolescent patient treated for catatonia.

**Methods:**

A 15-year-old boy with a background of severe dyspraxia but otherwise normal development and no prior health problems was admitted to an adolescent mental health unit due to rapid cognitive, motor, functional, and psychological decline, suggestive of catatonia. A paediatric immunology work up found no organic or autoimmune cause. An MRI head was unremarkable. A lorazepam challenge produced transient improvement. Due to persistent severe catatonia with emerging psychotic symptoms, an eight-session course of ECT was initiated under an STDC, later converted to a hospital CTO. Gradual recovery was observed, with Bush–Francis Catatonia Rating Scale scores reducing from 16 to 0. The patient regained normal motor coordination and speech, with improved emotional regulation and social interaction. Residual psychotic symptoms were mild, with preserved insight. Discharge plans included medication review, movement from inpatient to community, phased return to school, and gradual home passes. The patient was discharged from inpatient care after a 2.5 month stay.

**Results:**

Adolescent catatonia is frequently under-recognised and misattributed to neurological or functional disorders, leading to delays in effective treatment. This case is notable due to the rapid progression and severity of symptoms, with prominent motor, cognitive, and autonomic features closely mimicking organic pathology. Although benzodiazepines are recommended as first-line treatment, response in paediatric populations is often incomplete. This patient demonstrated partial improvement which necessitated escalation, illustrating the importance of early consideration of ECT, which is supported by growing evidence as safe and highly effective intervention for severe, or treatment-resistant paediatric catatonia, with reported response rates exceeding 70%. Additionally, the case highlights the ethical and legal complexities of compulsory treatment in young people, demonstrating how proportionate use of legislation can facilitate recovery and restoration of autonomy.

**Conclusion:**

Catatonia in adolescents is serious but highly treatable. Early recognition and escalation of treatment reduce morbidity and risk. Benzodiazepines are first-line, but clinicians should maintain a low threshold for ECT in cases of partial response or high clinical risk, given its safety and efficacy in young people. Treatment guided by clinical presentation can result in full functional recovery and improved outcomes.

